# Associations between electrocardiogram and carotid ultrasound parameters: a healthy chinese group study

**DOI:** 10.3389/fphys.2022.976254

**Published:** 2022-08-08

**Authors:** Lingwei Shi, Dongsheng Bi, Jingchun Luo, Wei Chen, Cuiwei Yang, Yan Zheng, Ju Hao, Ke Chang, Boyi Li, Chengcheng Liu, Dean Ta

**Affiliations:** ^1^ Center for Biomedical Engineering, School of Information Science and Technology, Fudan University, Shanghai, China; ^2^ Human Phenome Institute, Fudan University, Shanghai, China; ^3^ Academy for Engineering and Technology, Fudan University, Shanghai, China; ^4^ Shanghai Key Laboratory of Medical Image Computing and Computer Assisted Intervention, Fudan University, Shanghai, China

**Keywords:** cardiovascular disease (CVD), electrocardiogram (ECG), carotid ultrasound (CUS), resistance index (RI), ST-segment amplitude (STA), cohort study

## Abstract

**Background:** Electrocardiogram (ECG) and carotid ultrasound (CUS) are important tools for the diagnosis and prediction of cardiovascular disease (CVD). This study aimed to investigate the associations between ECG and CUS parameters and explore the feasibility of assessing carotid health with ECG.

**Methods:** This cross-sectional cohort study enrolled 319 healthy Chinese subjects. Standard 12-lead ECG parameters (including the ST-segment amplitude [STA]), CUS parameters (intima-media thickness [IMT] and blood flow resistance index [RI]), and CVD risk factors (including sex, age, and systolic blood pressure [SBP]) were collected for analysis. Participants were divided into the high-level RI group (average RI ≥ 0.76, *n* = 171) and the normal RI group (average RI < 0.76, *n* = 148). Linear and stepwise multivariable regression models were performed to explore the associations between ECG and CUS parameters.

**Results:** Statistically significant differences in sex, age, SBP, STA and other ECG parameters were observed in the normal and the high-level RI group. The STA in lead V_3_ yielded stronger significant correlations (r = 0.27–0.42, *p* < 0.001) with RI than STA in other leads, while ECG parameters yielded weak correlations with IMT (|r| ≤ 0.20, *p* < 0.05). STA in lead V_2_ or V_3_, sex, age, and SBP had independent contributions (*p* < 0.01) to predicting RI in the stepwise multivariable models, although the models for IMT had only CVD risk factors (age, body mass index, and triglyceride) as independent variables. The prediction model for RI in the left proximal common carotid artery (CCA) had higher adjusted R^2^ (adjusted R^2^ = 0.31) than the model for RI in the left middle CCA (adjusted R^2^ = 0.29) and the model for RI in the right proximal CCA (adjusted R^2^ = 0.20).

**Conclusion:** In a cohort of healthy Chinese individuals, the STA was associated with the RI of CCA, which indicated that ECG could be utilized to assess carotid health. The utilization of ECG might contribute to a rapid screening of carotid health with convenient operations.

## Introduction

Cardiovascular disease (CVD) is one of the four major non-communicable diseases, which caused 17.9 million deaths in 2019 (WHO, 2021). CVD risk increases with high body mass index (BMI) (≥25 kg/m^2^), hypertension, and smoking, which are also associated with atherosclerosis (Arnett et al., 2019; Liu et al., 2021). Atherosclerosis is the earliest occurrence of CVD and is characterized by plaque and increased intima-media thickness (IMT) in the innermost layer of carotid arteries (Handa et al., 1990; Palve et al., 2014; Libby et al., 2019; Lubrano and Balzan, 2021). Carotid plaque and high IMT increase the risk of myocardial infarction (Lorenz et al., 2007; Naqvi and Lee, 2014; Rafati et al., 2019). Myocardial infarction decreases the blood flow through the coronary artery, severely restricting the metabolism of cardiomyocytes and compromising myocardial function (Zhou et al., 2021). Therefore, carotid health assessment plays an essential role in CVD prevention.

Carotid ultrasound (CUS) is widely applied in carotid health screening, including plaque diagnosis and carotid parameter measurements (Handa et al., 1990; ter Avest et al., 2007; Latha et al., 2020). Plaque and IMT are assessed by B-mode ultrasound, while Doppler ultrasound can be used to measure hemodynamic parameters, including resistance index (RI). In addition to structural parameters (plaque, IMT), Doppler ultrasound contributes to predicting stroke and ischemic heart disease, independent of CVD risk factors and IMT (Chuang et al., 2016). Higher RI is associated with ischemic stroke independently of carotid atherosclerosis and CVD risk factors (Bai et al., 2007).

Standard 12-lead electrocardiogram (ECG) is the most common CVD diagnostic method, essential for diagnosing myocardial infarction and other CVDs (Kligfield et al., 2007; Wagner et al., 2009; Stracina et al., 2022). Myocardial infarction is associated with ST-segment elevation in standard 12-lead ECG (Alpert et al., 2000). In addition to myocardial infarction, ST-segment elevations may be caused by right bundle branch block, acute pericarditis, Brugada syndrome, and other diseases (Nishizaki et al., 2003; Hanna and Glancy, 2015). In case of ST-segment surpassing threshold value, precise diagnoses should consist of patient symptoms, ST-segment waveforms, involved leads, and other ECG characteristics. ST-segment elevation can also be observed even in healthy people (Wang et al., 2003; de Bliek, 2018), indicating other associations between ECG and phenotypes besides direct CVDs.

The ECG and CUS examinations contribute to the diagnosis of CVD with their own specialties and diagnostic performance. There might be some immanent correlations between the ECG and CUS parameters for the CVD diagnosis. However, few studies have investigated the associations between the ECG and CUS parameters in the diagnosis of CVD. Ulgen et al. (2001) suggested that coronary artery disease could lead to a decrease in carotid flow velocities and an increase in RI. The IMT is independently associated with heart rate variability in individuals with at least one CVD risk factor (Pereira et al., 2015; Pereira et al., 2017). Zeng et al. (2020) found that carotid atherosclerosis can be diagnosed by an ECG-based R wave pulse wave index. The associations between ECG and CUS parameters need to be studied. Therefore, the present study was designed to investigate the associations between ECG and CUS parameters and explore the feasibility of assessing carotid health with the ECG.

## Materials and methods

### Study design

**FIGURE 1:**
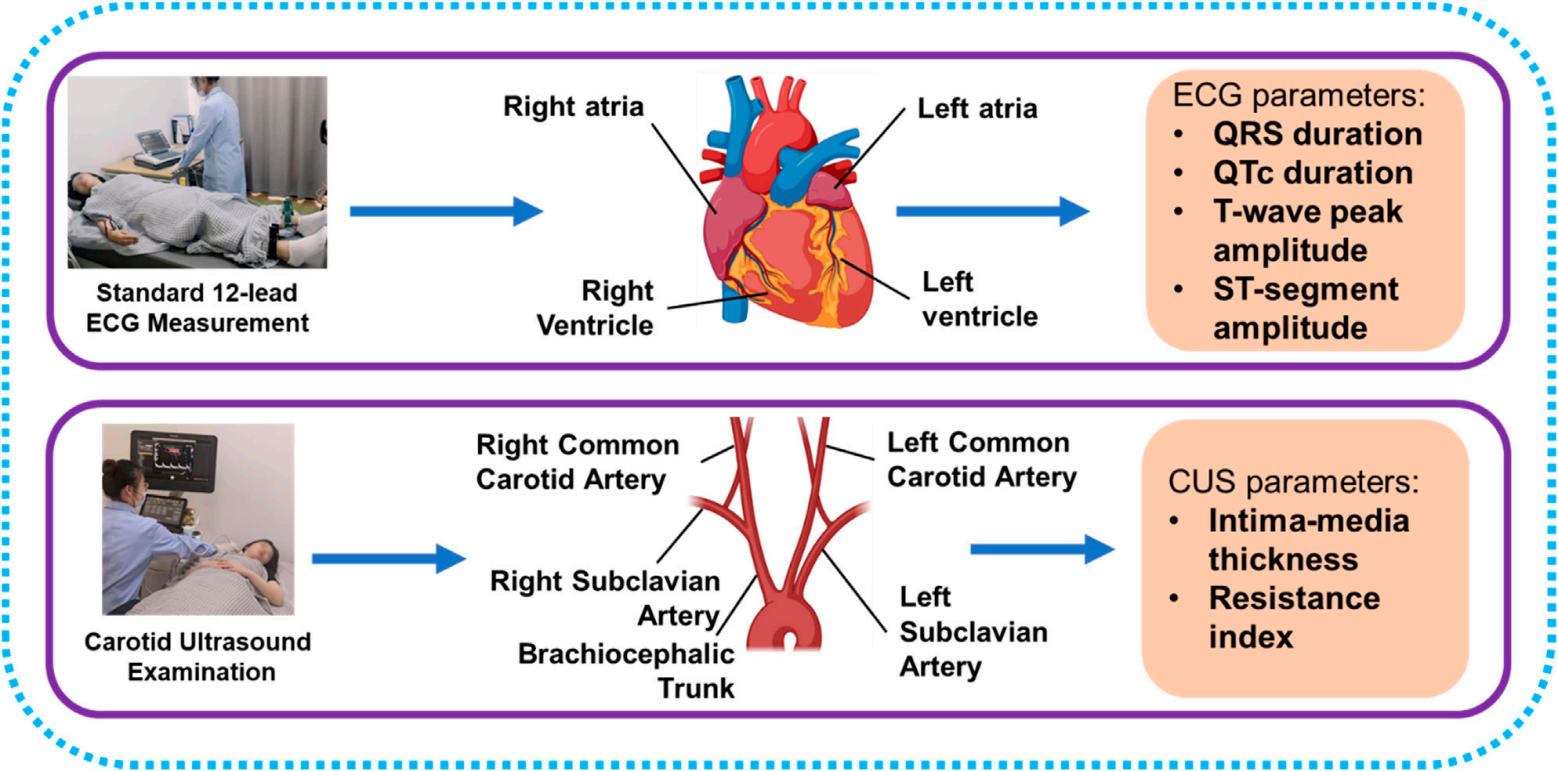
Process of ECG signals collecting and CUS examinations.

We proposed a cross-sectional observational study with a healthy Chinese group to investigate the associations between ECG and CUS parameters and the potential of ECG to evaluate carotid health. A total of 319 subjects participated in this study. None of the subjects had diabetes, hypertension, asthma, CVDs, tumor, or mental disorder. Processes of ECG and CUS parameter acquisitions are displayed in Figure 1, and the ECG and CUS parameters were measured within 3 hours. CVD risk factors were collected through physical examinations, fasting blood sampling, and questionnaires, including sex, age, BMI, systolic blood pressure (SBP), diastolic blood pressure (DBP), triglyceride (TG), high-density lipoprotein cholesterol (HDL-C), low-density lipoprotein cholesterol (LDL-C), total cholesterol (TC), fasting plasma glucose (FPG), and smoking. The experimental protocol was approved by the institutional review boards of the School of Life Sciences and Zhongshan Hospital of Fudan University. Informed consent for the study was obtained from all subjects.

### Carotid ultrasound examination

CUS examinations were performed using a color ultrasonic diagnosis system (EPIQ 7, Philips Ultrasound, Amsterdam, NLD) with a linear array transducer (No. L12-5, effective frequency: 5–12 MHz, central frequency: 6.9 MHz, and image depth: 1–4 cm). The examinations were performed with subjects in the supine position. All CUS examinations were conducted by a well-trained technician. Preset settings and the automatic adjustment of the ultrasonic diagnosis system were adopted to improve image quality, following the instruction provided by the manufacturer.

**FIGURE 2:**
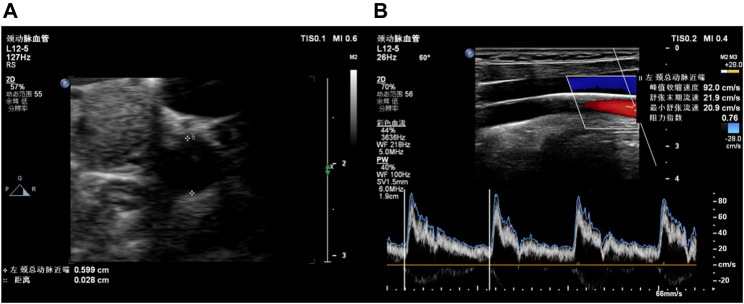
Examples of carotid ultrasound. **(A)** B-mode ultrasound for IMT; **(B)** Doppler ultrasound for RI.

B-mode ultrasound and Doppler ultrasound were used to assess carotid health (Figure 2). CUS examinations included three locations of the right and left common carotid arteries, i.e., proximal common carotid artery (PCCA), middle common carotid artery (MCCA), and distal common carotid artery (DCCA). PCCA was the beginning of the carotid artery, while DCCA was defined as 1 cm proximal to the bifurcation. MCCA is in the middle of PCCA and DCCA. At the beginning of the examination, the carotid artery was continuously imaged with B-mode ultrasound by pushing the transducer along the vessel. Plaques were found in five participants during imaging the whole vessel according to the diagnosis standard of carotid plaque (Touboul et al., 2012; Saba et al., 2019). Then B-mode ultrasound was used to measure IMT in PCCA, MCCA, and DCCA during diastole. RIs of PCCA, MCCA, and DCCA were measured by Doppler ultrasound in a cardiac cycle. RI was automatically calculated with peak systolic velocity and end diastolic velocity by the ultrasonic diagnosis system. The average RI values of bilateral sides were calculated. Chuang et al. (2016) analyzed the associations between carotid blood flow velocity and risk of future CVD, and found that people with RI higher than 0.76 have increased risk of CVDs. Based on average RI values, volunteers were divided into the normal RI group (average RI values of each side <0.76) and the high-level RI group (average RI value of one side ≥0.76).

### ECG parameter measurement

An ECG analyzer (MAC 5500, GE, Boston, MA, United States) was used to record ECG signals. ECG parameters were calculated automatically by the analyzer with standard 12-lead ECG signals. The analyzer was operated by a well-trained technician. ECG signals were not collected until volunteers remained supine and rested for 5 min. ECG parameters included in this study consist of QRS complex duration, QT interval duration, T-wave peak amplitude (TWPA), and ST-segment amplitude (STA). Since QT interval is interfered with by heart rate, correcting the influence of the heart rate on the QT interval is necessary. According to the recommendation of AHA/ACCF/HRS (Luo et al., 2004; Rautaharju et al., 2009), the QT interval was corrected based on Framingham linear equation: QTc=QT+154×(1−60/HR) , where QTc is the corrected QT interval duration, QT is the QT interval duration without correction in milliseconds, and HR is the heart rate (beats per minute, bpm). STA and TWPA include values collected from 12 leads. STA values were measured at the junction of the ST-segment and the QRS complex, i.e., “J point” (Rautaharju et al., 2009; Hanna and Glancy, 2015).

### Statistical analysis

All data analyses were conducted with Matlab 2020b (MathWorks, Natick, MA, United States). Anderson-Darling tests were used to examine the normality of all the variables. Results indicated that only eight in 49 parameters followed normal distributions (*p* < 0.05). The descriptive statistics of ECG parameters and CVD risk factors were depicted using the median and interquartile range in the normal and the high-level RI groups. Differences between the groups were determined using the Wilcoxon rank-sum test for continuous variables and the chi-square (χ^2^) test for categorical variables. Simple linear regressions (Spearman’s rank correlation) were used to evaluate the relationships between variables. Finally, stepwise multivariable regressions were used to produce models for predicting the CUS parameters using combinations of ECG parameters and CVD risk factors. A *p*-value less than 0.05 was considered statistical significance.

## Results

### Descriptive statistics for parameters of subjects

A total of 319 subjects were enrolled in our study, including 194 women and 125 men. The median age was 30 (interquartile range, 24–42). Descriptive statistics of CVD risk factors and several ECG parameters of subjects in the normal and high-level RI groups are listed in Table 1. Subjects in the high-level RI group were younger (28.0 [24.0–36.0] years) than those in the normal RI group (33.5 [25.0–49.5] years). The participants in the high-level RI group had higher STA in lead V_3_ (36.0 [9.0–68.0] μV) than in the normal RI group (68.0 [39.0–116.5] μV). In addition, there are significant differences in the man proportion, SBP, and other ECG parameters between the high-level RI group and the normal RI group. Descriptive statistics of CUS parameters of all subjects are summarized in Table 2.

**TABLE 1:** Statistics of CVD risk factors and ECG parameters for participants in the normal RI group and the high-level RI group.

Characteristics		All participants *N* = 319	RI		*p*-value
			Normal N = 148	High-level N = 171	
CVD Risk Factors	Age (years)	30.0 (24.0–42.0)	33.5 (25.0–49.5)	28.0 (24.0–36.0)	<0.001
	Men, n %	125	35 (23.6%)	90 (52.6%)	<0.001
	BMI (kg/m2)	22.3 (20.3–24.6)	22.2 (20.1–24.6)	22.3 (20.4–24.6)	0.84
	SBP (mmHg)	110 (100–120)	110 (100–116)	110 (100–120)	0.004
	DBP (mmHg)	70 (64–80)	70 (63–78)	70.0 (65.0–80.0)	0.15
	TG (mmol/L)	0.85 (0.65–1.25)	0.81 (0.62–1.17)	0.88 (0.68–1.29)	0.24
	HDL-C (mmol/L)	1.46 (1.25–1.71)	1.50 (1.27–1.71)	1.44 (1.23–1.71)	0.42
	LDL-C (mmol/L)	2.41 (1.92–2.85)	2.33 (1.87–2.76)	2.46 (1.99–2.94)	0.17
	TC (mmol/L)	4.33 (3.85–4.94)	4.29 (3.83–4.83)	4.40 (3.96–4.96)	0.16
	FPG (mmol/L)	4.78 (4.50–5.00)	4.80 (4.53–5.10)	4.70 (4.49–5.00)	0.07
	Smoking, n %	14 (4.4%)	7 (4.7%)	7 (4.1%)	0.78
ECG parameters	QRS Duration (ms)	90.0 (82.5–96.0)	88.0 (82.0–94.0)	92.0 (84.5–98.0)	<0.001
	QTc Duration (ms)	411 (398–424)	417 (405–430)	405 (394–418)	<0.001
	TWPA in Lead I (μV)	209 (157–258)	188 (142–244)	220 (172–273)	<0.001
	TWPA in Lead aVR (μV)	−278 (-334–-207)	−253 (−315–−192)	−291 (−356–-239)	<0.001
	TWPA in Lead V2 (μV)	507 (333–750)	410 (273–614)	610 (388–854)	<0.001
	TWPA in Lead V3 (μV)	522 (347–723)	423 (282–596)	620 (438–809)	<0.001
	STA in Lead I (μV)	19.0 (9.0–34.0)	19.0 (4.0–30.0)	22.0 (10.5–39.0)	0.01
	STA in Lead aVR (μV)	−35.0 (-44.0–-20.0)	−29.0 (−43.0–17.0)	−40.0 (−53.5–-25.0)	<0.001
	STA in Lead V2 (μV)	51.0 (14.0–102.0)	36.0 (4.5–85.0)	74.0 (32.0–121.0)	<0.001
	STA in Lead V3 (μV)	51.0 (24.0–92.0)	36.0 (9.0–68.0)	68.0 (39.0–116.5)	<0.001

Values represent N (%) or median (interquartile range). Normal RI, average RI values of each side <0.76; High-level RI, average RI value of one side ≥0.76. RI, resistance index; BMI, body mass index; SBP, systolic blood pressure; DBP, diastolic blood pressure; TG, triglyceride; HDL-C, high-density lipoprotein cholesterol; LDL-C, low-density lipoprotein cholesterol; TC, total cholesterol; FPG, fasting plasma glucose; TWPA, T-wave peak amplitude; STA, ST-segment Amplitude.

**TABLE 2:** Statistics of CUS parameters for participants in the normal RI group and the high-level RI group.

Characteristics	All participants N = 319	RI		*p*-value
		Normal N = 148	High-level N = 171	
RI in Left PCCA	0.79 (0.75–0.82)	0.76 (0.72–0.78)	0.81 (0.79–0.84)	<0.001
RI in Right PCCA	0.79 (0.76–0.82)	0.77 (0.74–0.78)	0.82 (0.79–0.84)	<0.001
RI in Left MCCA	0.75 (0.71–0.79)	0.72 (0.69–0.73)	0.78 (0.76–0.81)	<0.001
RI in Right MCCA	0.75 (0.71–0.79)	0.71 (0.69–0.74)	0.79 (0.76–0.81)	<0.001
RI in Left DCCA	0.72 (0.68–0.75)	0.68 (0.65–0.71)	0.74 (0.72–0.77)	<0.001
RI in Right DCCA	0.71 (0.67–0.75)	0.67 (0.64–0.70)	0.74 (0.71–0.77)	<0.001
IMT in Left PCCA (cm)	0.04 (0.04–0.05)	0.04 (0.04–0.05)	0.04 (0.04–0.05)	0.08
IMT in Right PCCA (cm)	0.05 (0.04–0.06)	0.05 (0.04–0.06)	0.05 (0.04–0.05)	0.45
IMT in Left MCCA (cm)	0.05 (0.04–0.05)	0.05 (0.04–0.05)	0.05 (0.04–0.05)	0.72
IMT in Right MCCA (cm)	0.05 (0.04–0.05)	0.05 (0.04–0.06)	0.04 (0.04–0.05)	0.03
IMT in Left DCCA (cm)	0.05 (0.04–0.06)	0.05 (0.04–0.06)	0.05 (0.04–0.06)	0.49
IMT in Right DCCA (cm)	0.05 (0.04–0.06)	0.05 (0.04–0.06)	0.05 (0.04–0.06)	0.91

Values represent median (interquartile range). RI, resistance index; PCCA, proximal common carotid artery; MCCA, middle common carotid artery; DCCA, distal common carotid artery; IMT, intima-media thickness.

### Associations among ECG, CUS, and CVD risk factors

The correlations between CUS parameters and CVD risk factors are listed in Table 3. RI yielded significant correlations with sex, age and SBP (|r| ≤ 0.34, *p* < 0.001). In addition, age, BMI, SBP, DBP, TG, LDL-C, TC, FPG, and smoking were significant factors influencing IMT (|r| ≤ 0.48, *p* < 0.001).

**TABLE 3:** Spearman correlations between CUS parameters and CVD risk factors.

	Sex	Age	BMI	SBP	DBP	TG	HDL-C	LDL-C	TC	FPG	Smoking
RI in Left PCCA	0.26^§^	−0.37^§^	-	0.17^‡^	-	-	-	-	-	−0.13^†^	-
RI in Right PCCA	0.19^§^	−0.27^§^	-	0.15^‡^	-	-	-	-	-	-	-
RI in Left MCCA	0.34^§^	−0.24^§^	-	0.17^‡^	0.12^†^	-	-	-	-	−0.12^†^	-
RI in Right MCCA	0.26^§^	−0.30^§^	-	0.15^‡^	-	-	−0.12^†^	-	-	-	-
RI in Left DCCA	0.30^§^	−0.17^‡^	-	0.21^§^	0.12^†^	-	-	-	-	-	-
RI in Right DCCA	0.29^§^	−0.17^‡^	0.12^†^	0.22^§^	0.15^‡^	-	-	-	-	-	-
IMT in Left PCCA	-	0.29^§^	0.24^§^	0.17^‡^	0.11^†^	-	-	0.19^§^	0.17^‡^	0.20^§^	-
IMT in Right PCCA	-	0.32^§^	0.14^†^	0.16^‡^	0.17^‡^	-	-	0.12^†^	0.11^†^	-	-
IMT in Left MCCA	0.15^‡^	0.45^§^	0.27^§^	0.18^‡^	0.19^§^	0.24^§^	−0.16^‡^	0.28^§^	0.24^§^	0.22^§^	-
IMT in Right MCCA	0.14^†^	0.48^§^	0.23^§^	0.19^§^	0.18^‡^	0.21^§^	−0.15^‡^	0.20^§^	0.16^‡^	0.18^§^	0.13^†^
IMT in Left DCCA	-	0.44^§^	0.23^§^	0.13^†^	-	0.15^‡^	-	0.23^§^	0.24^§^	0.16^‡^	0.12^†^
IMT in Right DCCA	0.13^†^	0.45^§^	0.19^§^	0.16^‡^	0.14^†^	0.19^§^	-	0.22^§^	0.25^§^	0.19^§^	0.13^†^

PCCA, proximal common carotid artery; MCCA, middle common carotid artery; DCCA, distal common carotid artery; RI, resistance index; IMT, intima-media thickness; BMI, body mass index; SBP, systolic blood pressure; DBP, diastolic blood pressure; TG, triglyceride; HDL-C, high-density lipoprotein cholesterol; LDL-C, low-density lipoprotein cholesterol; TC, total cholesterol; FPG, fasting plasma glucose.

-, not significant; †, *p* < 0.05; ‡, *p* < 0.01; §, *p* < 0.001.

**FIGURE 3:**
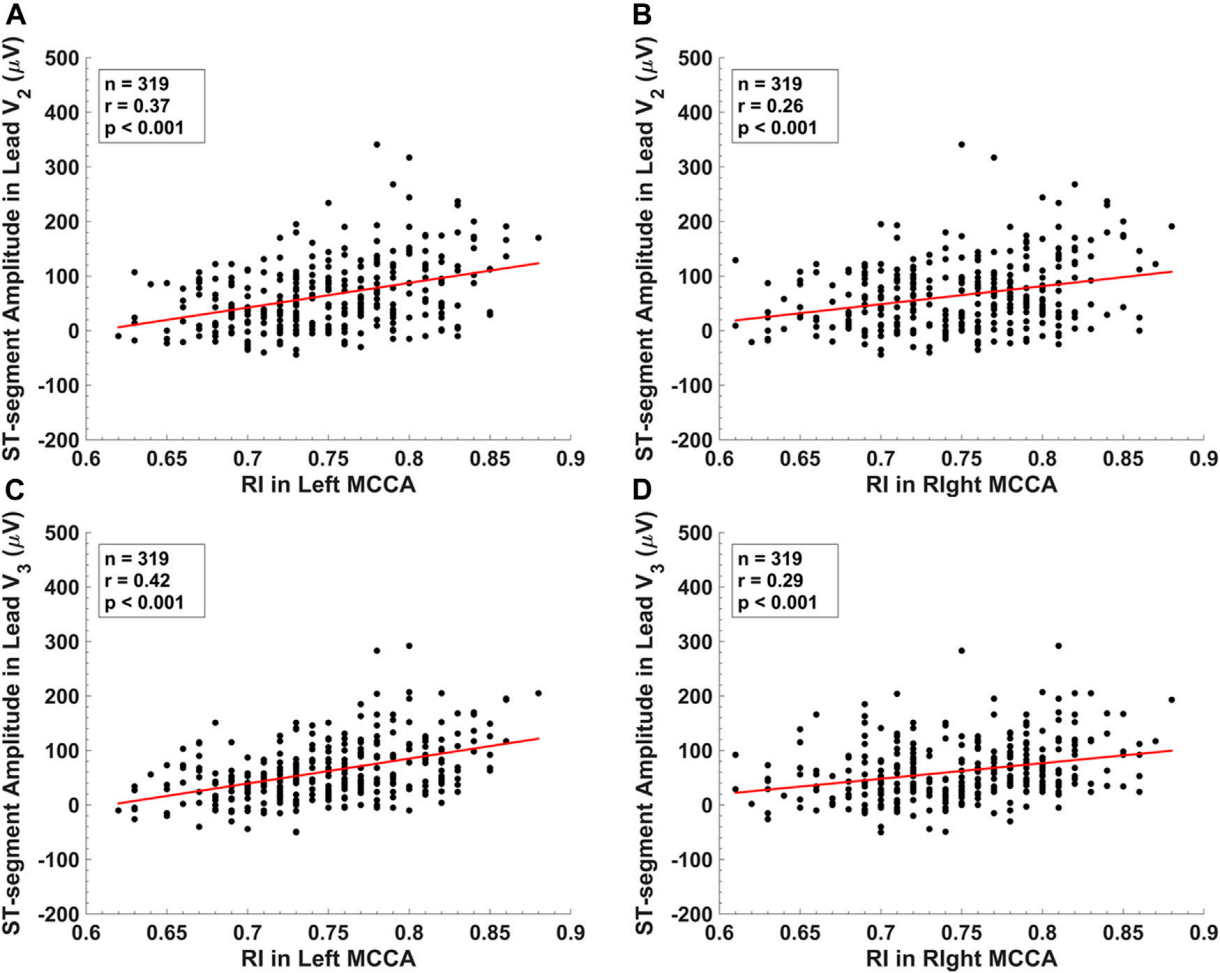
The scatter plots between **(A)** ST-segment amplitude in lead V_2_ and RI in the left middle common carotid artery. **(B)** ST-segment amplitude in lead V_2_ and RI in the right middle common carotid artery. **(C)** ST-segment amplitude in lead V_3_ and RI in the left middle common carotid artery. **(D)** ST-segment amplitude in lead V_3_ and RI in the right middle common carotid artery.

Figure 3 displays the scatterplots between the STAs in lead V_2_, lead V_3_ and RIs of the left MCCA, the right MCCA. The STA in lead V_2_ yielded a significant correlation with RI in the left MCCA (r = 0.37, *p* < 0.001) and RI in the right MCCA (r = 0.26, *p* < 0.001). Significant correlations of the STA in lead V_3_ were observed with RI in the left MCCA (r = 0.42, *p* < 0.001) and RI in the right MCCA (r = 0.29, *p* < 0.001). Table 4 summarizes the correlations between CUS and ECG parameters. Significant correlations were observed between the RIs and ECG parameters (|r| ≤ 0.42, *p* < 0.05). ECG parameters had stronger correlations with RIs in left CCA than RIs at the exact locations in right CCA. The RI in the right MCCA yielded the strongest correlation with QTc (r = -0.33, *p* < 0.001), while the rest RIs yielded the strongest associations with the STA in lead V_2_ or lead V_3_. A few carotid IMTs yielded significant correlations with ECG parameters (|r| ≤ 0.20, *p* < 0.05).

**TABLE 4:** Spearman correlations between ECG parameters and CUS parameters.

	IMT in left DCCA	RI in right PCCA	RI in left MCCA	RI in right MCCA	IMT in left PCCA	IMT in left PCCA	IMT in left DCCA	IMT in left DCCA
QRS Duration	0.21^§^	0.17^‡^	0.26^§^	0.22^§^	-	-	-	-
QTc Duration	−0.33^§^	−0.26^§^	−0.30^§^	−0.33^§^	-	0.15‡	-	0.11^†^
TWPA in Lead II	0.23^§^	0.20^§^	0.15^‡^	0.16^‡^	-	-	-	−0.20^§^
TWPA in Lead aVF	0.20^§^	0.18^‡^	-	0.13^†^	-0.12†	-	-	−0.19^§^
TWPA in Lead V_2_	0.30^§^	0.24^§^	0.35^§^	0.26^§^	-	-	-	-
TWPA in Lead V_5_	0.22^§^	0.16^‡^	0.18^‡^	0.15^‡^	−0.12†	-	-	−0.11^†^
STA in Lead II	0.25^§^	0.20^§^	0.18^§^	0.20^§^	−0.12†	−0.19§	-	−0.15^‡^
STA in Lead aVR	−0.24^§^	−0.19^§^	−0.22^§^	−0.22^§^	-	0.15‡	-	0.14^†^
STA in Lead aVF	0.22^§^	0.16^‡^	0.13^†^	0.16^‡^	−0.13†	−0.19§	-	−0.13^†^
STA in Lead V_2_	0.29^§^	0.25^§^	0.37^§^	0.26^§^	-	−0.11†	-	-
STA in Lead V_3_	0.39^§^	0.29^§^	0.42^§^	0.29^§^	-	−0.16‡	-	-
STA in Lead V_4_	0.36^§^	0.28^§^	0.38^§^	0.27^§^	-	−0.15‡	-	−0.13^†^

PCCA, proximal common carotid artery; MCCA, middle common carotid artery; DCCA, distal common carotid artery; RI, resistance index; IMT, intima-media thickness; TWPA, T-wave peak amplitude; STA, ST-segment Amplitude.

-, not significant; †, *p* < 0.05; ‡, *p* < 0.01; §, *p* < 0.001.

### Stepwise multivariable regression for predicting CUS parameters

Stepwise multivariable regressions were performed to produce CUS parameter prediction models with ECG parameters and CVD risk factors. ECG parameters had independent contributions to stepwise models for predicting RIs (Table 5). The stepwise multivariable regression model with the STA in lead V_3_, sex, age, and SBP as independent variables (*p* < 0.01) can explain up to 31% of the variation of RI in the left PCCA. The independent variables of the stepwise regression prediction model for RI in the left MCCA were the STA in lead V_3_, sex, and age (adjusted R^2^ = 0.29, *p* < 0.001). The combination of the STA in lead V_2_, age, and SBP (*p* < 0.01) can explain up to 20% of the variation of RI in the right PCCA. CVD risk factors contributed significantly in the stepwise prediction models for IMT (Table 5).

**TABLE 5:** Stepwise Multivariable regression models for predicting CUS parameters with ECG parameters and CVD risk factors as independent variables.

CUS Dependent variable	Independent variables	RMSE	Adjusted R^2^
RI in Left PCCA	STA in Lead V_3_^‡^, Sex^‡^, Age^§^, SBP^‡^	0.040	0.31
RI in Right PCCA	STA in Lead V_2_^§^, Age^§^, SBP^§^	0.043	0.20
RI in Left MCCA	STA in Lead V_3_^§^, Sex^§^, Age^§^	0.044	0.29
IMT in Left PCCA	Age^§^, BMI^§^	0.008	0.12
IMT in Right PCCA	Age^§^	0.009	0.12
IMT in Left MCCA	Age^§^, TG^§^	0.008	0.25

CUS, carotid ultrasound; PCCA, proximal common carotid artery; MCCA, middle common carotid artery; RI, resistance index; IMT, intima-media thickness; STA, ST-segment amplitude; SBP, systolic blood pressure; BMI, body mass index; TG, triglyceride.

†, *p* < 0.05; ‡, *p* < 0.01; §, *p* < 0.001.

## Discussion

This cross-sectional cohort study investigated the associations between ECG and CUS parameters in healthy Chinese individuals. Significant associations between STA and RI were observed, which were demonstrated to be independent of CVD risk factors. RI yielded strong correlations with ECG parameters, while IMT yielded little correlations with ECG parameters. In addition, RI prediction models were established with ECG parameters and CVD risk factors. The STAs in V_3_ and V_2_ significantly contributed to the stepwise models for predicting RIs. These results proved that there are significant associations between ECG and CUS parameters, and ECG could be utilized in the assessment of carotid health.

ECG records the cardiac electrical activities of atriums and ventricles. During the electrical activities of ventricles, blood is pumped from ventricles into the systemic circulation. The carotid artery is an essential part of the systemic circulation. We suspected that there were close associations between the carotid and ECG signals during ventricular activity and analyzed the CUS parameters and relevant ECG parameters. Considering the recommendations of AHA/ACCF/HRS in 2009 (Rautaharju et al., 2009), ECG parameters consisted of QRS duration, QTc duration, STA, and TWPA in this study. The results found that the ECG parameters had significant associations with CUS parameters.

In our study, compared with the IMT, the RI had relatively stronger associations with ECG. The ECG parameters and RI yielded significant correlations (r up to 0.42). This study consisted of healthy Chinese individuals. This might influence the correlation coefficients between ECG and CUS parameters. Carotid IMT is an essential indicator of cardiovascular health. Many studies have demonstrated that increased carotid IMT significantly correlates with CVDs (Touboul et al., 2007; Coskun et al., 2009; Cui et al., 2020). However, carotid RI is a parameter reflecting the blood flow of carotid arteries. In addition, ECG records the electrical activities of the cardiac which is the most crucial organ in the circulatory system and determines the blood flow of the carotid artery. In other words, the cardiac activities, reflected by ECG, determine the blood flow in the systemic circulation. This might be the reason for the significant correlations between RI and ECG, which are stronger than the correlations between IMT and ECG.

This study found that RI yielded stronger correlations with the STAs in lead V_2_ and lead V_3_ than ECG parameters in other leads. In Table 5, only the STA in lead V_2_ or lead V_3_ had significant contributions to predicting RI in the stepwise multivariable regression models. Lead V_1_-V_6_ are precordial leads and others are limb leads (i.e., lead I, II, III, aVR, aVL, and aVF). In addition, lead V_2_ and lead V_3_ are the closest leads to the left ventricle which pumps blood to the systemic circulation. Carotid arteries are in the systemic circulation and carotid RI is determined by the blood flow from left ventricle. These might be reasons why carotid in the systemic circulation is more closely related to lead V_2_ and lead V_3_ than other leads.

The locations for RI measuring influenced the associations between RI and STA. Compared with RIs in right CCA, RIs in left CCA yielded stronger correlations with ECG parameters (Table 4). According to Table 5, RI in the left CCA and RI in PCCA had better prediction effects in the stepwise regression models than RI in the right CCA and RI in MCCA, respectively. The left carotid artery is directly connected to the aorta, while the right carotid artery is connected to the aorta through the brachiocephalic trunk supplying blood to the right arm as well. The correlations between RIs in right CCA and ECG parameters might be influenced by the blood flow to right arm. Moreover, compared with the MCCA, the PCCA is adjacent to and more closely associated with the heart, which resulted in better predictions. The RIs measured far from the heart might be influenced by carotid artery structures in addition to the determination of heart. Therefore, direct connection between the aorta and left carotid artery led to a stronger association between RI in the left PCCA and STAs and a more precise prediction of the stepwise multivariable regression model for RI in the left PCCA.

It is generally believed that the health of the carotid artery will deteriorate with age. A consequence of the deterioration is the increase of RI, but it was observed in our healthy individual cohort study that the age of subjects in the high-level RI group was significantly younger than the normal RI group. This result might be explained by sex and STA reasonably. In healthy populations, the STA of young men is higher than that of aged men, while the STA of women does not change with age (Wang et al., 2003; Wagner et al., 2009; Hanna and Glancy, 2015; de Bliek, 2018). Compared with the normal RI group, the high-level RI group had more male participants and higher STA, corresponding to the younger age in the high-level RI group than the normal RI group. RI is a hemodynamic parameter that measures carotid blood flow resistance. The increased resistance in the carotid may lead to increased left heart pressure, enlargement of the left atrium and left ventricle, and increased STA. Therefore, correlation coefficients between the STAs and RIs were positive.

In addition to age, sex and SBP were included in the stepwise multivariable regression models for predicting RIs. Sex and SBP had significant differences (*p <* 0.01) between the normal and the high-level RI groups. RI yielded significant correlations with sex and age (Table 3). Male volunteers or volunteers with high SBP tended to have higher RI. However, RI yielded little correlations with biomarker CVD risk factors. It is believed that biomarker CVD risk factors significantly influence IMT (Amarenco et al., 2008; Wurtz et al., 2012; Virtanen et al., 2016), a distinct difference between IMT and RI. These phenomena were in consistent with our findings for the correlation between CUS and biomarker CVD risk factors (table 3). Hemodynamic parameter RI was notably influenced by heart health, age, sex, and SBP. People of the male sex, young age, and high blood pressure might easily have increased RI, which could relate to potential CVDs. This result might contribute to CVD prevention in young populations.

In this cross-sectional study, we assessed the associations between ECG and CUS parameters, including QRS duration, STA, TWPA, QTc duration, carotid IMT, and RI, along with several CVD risk factors. This study demonstrated the independent associations between the ECG and CUS parameters and revealed ECG’s feasibility in assessing carotid health. We found that RI yielded strong associations with STA and can be predicted by the stepwise regression models consisting of STA and CVD risk factors. Another strength is that B-mode ultrasound and Doppler ultrasound were conducted at six locations of bilateral common carotid arteries to measure CUS parameters. ECG measurement is a common, fast, and convenient method to screen cardiac health and diagnose CVDs. We suggest further investigating the associations between ECG and CUS to establish the methods for assessing carotid health with ECG parameters.

The limitation of this study is that the study cohort consisted of only healthy individuals. The presence of coronary artery diseases could increase the value of RI (Ulgen et al., 2001). Pereira et al. (2017) found that increased IMT was associated with decreased heart rate variability values among subjects at increased CVD risk. This cross-sectional study indicated that ECG parameters are associated with CUS parameters among healthy Chinese individuals. We found that carotid health might be evaluated during ECG examinations. However, investigating associations between ECG and CUS parameters in a natural population (with and without CVDs) is necessary for assessing carotid health with ECG.

## Conclusions

This cross-sectional cohort study analyzed the ECG, CUS, and CVD risk factors of 319 subjects from a healthy Chinese group. The resistance index yielded significant correlations with the ST-segment amplitude independent of CVD risk factors. Our study proved that there are independent associations between ECG and CUS parameters, and ECG might have potential in the assessment of carotid health.

## Data Availability

The original contributions presented in the study are included in the article/Supplementary Material, further inquiries can be directed to the corresponding authors.
